# Coordinated local RNA overexpression of complement induced by interferon gamma in myositis

**DOI:** 10.1038/s41598-023-28838-z

**Published:** 2023-02-04

**Authors:** Maria Casal-Dominguez, Iago Pinal-Fernandez, Katherine Pak, Sandra Muñoz-Braceras, Jose C. Milisenda, Jiram Torres-Ruiz, Stefania Dell′Orso, Faiza Naz, Gustavo Gutierrez-Cruz, Yaiza Duque-Jaimez, Ana Matas-Garcia, Laura Valls-Roca, Gloria Garrabou, Ernesto Trallero-Araguas, Brian Walitt, Lisa Christopher-Stine, Thomas E. Lloyd, Julie J. Paik, Jemima Albayda, Andrea Corse, Josep Maria Grau, Albert Selva-O’Callaghan, Andrew L. Mammen

**Affiliations:** 1 Muscle Disease Unit, National Institute of Arthritis and Musculoskeletal and Skin Diseases, National Institutes of Health, 50 South Drive, Room 1141, Building 50, MSC 8024, Bethesda, MD 20892 USA; 2 Department of Neurology, Johns Hopkins University School of Medicine, Baltimore, MD USA; 3 Muscle Research Unit, Internal Medicine Service, Hospital Clinic, Barcelona, Spain; 4 Barcelona University, Barcelona, Spain; 5 Department of Immunology and Rheumatology, Instituto Nacional de Ciencias Médicas y Nutrición Salvador Zubirán, Mexico City, Mexico; 6 CIBERER, IDIBAPS, Barcelona, Spain; 7 Systemic Autoimmune Disease Unit, Vall d’Hebron Institute of Research, Barcelona, Spain; 8 Autonomous University of Barcelona, Barcelona, Spain; 9 National Institute of Neurological Disorders and Stroke, National Institutes of Health, Bethesda, MD USA; 10 Department of Medicine, Johns Hopkins University School of Medicine, Baltimore, MD USA

**Keywords:** Autoimmunity, Complement cascade, Immunology, Immunological disorders, Autoimmune diseases

## Abstract

Complement proteins are deposited in the muscles of patients with myositis. However, the local expression and regulation of complement genes within myositis muscle have not been well characterized. In this study, bulk RNA sequencing (RNAseq) analyses of muscle biopsy specimens revealed that complement genes are locally overexpressed and correlate with markers of myositis disease activity, including the expression of interferon-gamma (IFN*γ*)-induced genes. Single cell and single nuclei RNAseq analyses showed that most local expression of complement genes occurs in macrophages, fibroblasts, and satellite cells, with each cell type expressing different sets of complement genes. Biopsies from immune-mediated necrotizing myopathy patients, who have the lowest levels of IFN*γ*-induced genes, also had the lowest complement gene expression levels. Furthermore, data from cultured human cells showed that IFN*γ* upregulates complement expression in macrophages, fibroblasts, and muscle cells. Taken together, our results suggest that in myositis muscle, IFN*γ* coordinates the local overexpression of complement genes that occurs in several cell types.

## Introduction

The complement system is a set of soluble proteins that are part of the innate immune defense, connecting innate and adaptive immunity. There are nine central complement components (C1–C9) as well as numerous complement activators and regulators^1^. Complement proteins are mainly produced in the liver. However, many other cell types can produce complement proteins, in some cases after stimulation by cytokines^2^. Importantly, dysregulated activation of the complement system may contribute to autoimmune diseases like type II glomerulonephritis, age-related macular degeneration, and atypical hemolytic uremic syndrome^1,3^.

Complement deposition has been proposed to play a key pathogenic role in several neuromuscular diseases including the inflammatory myopathies (IM), a heterogenous family of muscle diseases that includes dermatomyositis (DM), the antisynthetase syndrome (AS), immune-mediated necrotizing myopathy (IMNM)^4–6^, and inclusion body myositis (IBM). While each type of IM has its own characteristic clinical, serological, and muscle biopsy features, the deposition of complement proteins has been described in muscle tissues from each. For example, muscle biopsies from DM patients reveal C3b, C4b, and C5b-9 (the membrane attack complex; MAC) on endomysial capillaries^7,8^. In contrast, IMNM biopsies include MAC deposition on necrotic muscle fibers, atrophic muscle fibers, small arteries, veins, and capillaries within the muscle tissue^6,9–12^.

Although a recent study using bulk RNAseq data showed that C1QB and C1QC genes are overexpressed in DM muscle tissue^13^, it remains unclear which cells within DM muscle tissue contribute to complement gene overexpression. Indeed, a comprehensive analysis of complement gene expression within each type of IM muscle tissue has not been described. In the current study, our objectives were to (a) quantify the local expression of complement genes in different types of IM, (b) study the association between complement expression and IM disease activity, (c) identify the types of cells expressing each complement gene in IM muscle, and (d) determine how local complement gene expression might be regulated within IM muscle tissue.

## Methods

### Patients

In this study, we included all muscle biopsies from patients enrolled in institutional review board-approved (IRB) longitudinal cohorts from the National Institutes of Health in Bethesda, MD; the Johns Hopkins Myositis Center in Baltimore, MD; the Vall d’Hebron Hospital, and the Clinic Hospital in Barcelona if they fulfilled Lloyd’s criteria for IBM^14^, or they fulfilled the Casal and Pinal criteria for other types of IM^15^, and were positive for one of the following myositis specific autoantibodies (MSA): anti-Jo1, anti-NXP2, anti-Mi2, anti-TIF1g, anti-MDA5, anti-SRP or anti-HMGCR. Autoantibody testing was performed using one or more of the following techniques: ELISA, immunoprecipitation of proteins generated by in vitro transcription and translation (IVTT-IP), line blotting (EUROLINE myositis profile), or immunoprecipitation from ^35^S-methionine-labeled HeLa cell lysates. We classified the patients as antisynthetase syndrome if they were positive for anti-Jo1 autoantibodies, as DM if they tested positive for anti-Mi2, anti-NXP2, anti-MDA5, or anti-TIF1g, and as IMNM if they had autoantibodies against SRP or HMGCR. We obtained histologically normal muscle biopsies to use as healthy comparators from the Johns Hopkins Neuromuscular Pathology Laboratory (n = 12), the Skeletal Muscle Biobank of the University of Kentucky (n = 8), and the National Institutes of Health (n = 13).

### Standard protocol approvals and patient consent

This study was approved by the Institutional Review Boards of the National Institutes of Health, the Johns Hopkins, the Clinic, and the Vall d’Hebron Hospitals. Written informed consent was obtained from each participant. All methods were performed in accordance with the relevant guidelines and regulations.

### RNA sequencing

Bulk RNAseq was performed on frozen muscle biopsy specimens as previously described^16–19^. Briefly, RNA was extracted with TRIzol (Thermo Fisher Scientific). Libraries were either prepared with the NeoPrep system according to the TruSeqM Stranded mRNA Library Prep protocol (Illumina, San Diego, CA), or with the NEBNext Poly(A) mRNA Magnetic Isolation Module and Ultra™ II Directional RNA Library Prep Kit for Illumina (New England BioLabs, ref. #E7490 and #E7760).

### Single-nuclei RNA-sequencing

For the nuclei isolation, we used a modification of the sucrose-gradient ultracentrifugation nuclei isolation protocol from Schirmer et al.^20^ Ten mg of frozen muscle tissue was sectioned and homogenized in 1 mL of lysis buffer (0.32 M sucrose, 5 mM CaCl2, 3 mM MgCl2, 0.1 mM EDTA, 10 mM Tris–HCl pH 8, 1 mM DTT, 0.5% Triton X-100 in DEPC-treated water) using 1.4 mm ceramic beads low-binding tubes and the Bertin Technology Precellys 24 lysis homogenizer (6500 rpm-3times × 30 s). The homogenized tissue was transferred into open-top thick-walled polycarbonate ultracentrifuge tubes (25 × 89 mm, Beckman Coulter) on ice. 3.7 mL of sucrose solution (1.8 M sucrose, 3 mM MgCl2, 1 mM DTT, 10 mM Tris–HCl) were pipetted to the bottom of the tube containing lysis buffer generating two separated phases (sucrose on the bottom and homogenate on the top). The tubes were filled almost completely with lysis buffer and weighted for balance. The samples were ultracentrifuged (Beckman Coulter XE-90, SW32 rotor, swinging bucket) at 24,400 rpm (107,163rcf) for 2.5 h at 4 °C, transferred to ice, and the supernatant removed. Two hundred microliters of DEPC water-based PBS were added to each pellet, incubated on ice for 20 min, and then pellets were resuspended. The resulting samples were filtered twice using 30 μm Miltenyi pre-separation filters. The nuclei were counted using a manual hemocytometer. Between 2000 and 3000 nuclei per sample were loaded in the 10× Genomic Single-Cell 3’ system. We performed the 10× nuclei capture and the library preparation protocol according to the manufacturer’s instructions without modification.

### Single-cell RNA-sequencing

The cell isolation from human muscle biopsies was performed as follows: ~ 20–25 mg of fresh muscle tissue was placed in a 10 cm culture dish with 2 mL of dissociation buffer (10 ml of Ham-F 10% Horse serum, 1% Penicillin/streptomycin, and 51.28 mg of collagenase II (1000U/ml) (Gibco, ref. 1710-015) per sample. Muscle was minced with scissors into ~ 1-mm cubes, placed in 50 mL tubes with 10 mL of dissociation buffer, and incubated at 37 °C in a rocking water bath at 70–75 rpm for 50 min. After the incubation, washing media (Ham-F, 10% Horse serum, 1% penicillin/streptomycin) was added to the samples to bring the volume to 50 mL, and these were centrifuged for 5 min at 1500 rpm. Forty-two mL of the resulting supernatant was discarded and the remaining volume was used to triturate the pellet with a 5 mL pipet. One mL of Collagenase II (Gibco, ref. 17101-015) and 1 ml of 11U Dispase (Gibco, ref. 17105-041) was then added to the mixture and incubated at 37 °C in a rocking water bath at 70–75 rpm for 15 min. Next, the samples were mixed 10 times with a 10 mL syringe and a 20G needle. Washing medium was added to bring the volume to 50 mL and then the samples were centrifuged for 5 min at 1500 rpm. The resulting pellets were suspended in 10 mL of washing media and then filtered through a 70 μm strainer. 50 mL of washing media was added to each sample and these were centrifuged at 1500 rpm for 5 min. Next, supernatants were aspirated, leaving 300 μL of sample which was used for Fluorescent Activated Cell Sorting (FACS). A target capture of 10,000 cells per sample was loaded in the 10× Genomic Single-Cell 3’ system. We performed 10× nuclei capture as well as library preparation protocol according to the manufacturer’s recommendation without modification.

### Culture of differentiating human skeletal muscle myoblasts and treatment with different types of interferon

Normal human skeletal muscle myoblasts (HSMMs) were cultured according to the protocol recommended by the supplier (Lonza). When 80% confluent, the cultures were induced to differentiate into myotubes by replacing the growth medium with differentiation medium (Dulbecco’s modified Eagle’s medium supplemented with 2% horse serum and L-glutamine). Two plates of cells were harvested before differentiation and then daily for 6 days.

To examine the effect of different types of interferon on complement expression we treated HSMMs daily with 100 U/L and 1000 U/L of IFNA2a (R&D, ref. 11100-1), IFNB1 (PeproTech, ref. 300-02BC), and IFNG (PeproTech, ref. 300-02), respectively, for 7 days. Treated cells were harvested for RNA extraction and subsequent RNA sequencing.

### Statistical and bioinformatic analysis

Complement gene lists were obtained from the HUGO Gene Nomenclature Committee (HGNC). For the bulk RNAseq, reads were demultiplexed using bcl2fastq/2.20.0 and preprocessed using fastp/0.21.0. The abundance of each gene was generated using Salmon/1.5.2 and quality control output was summarized using multiqc/1.11. Counts were normalized using the Trimmed Means of M values (TMM) from edgeR/3.34.1 for graphical analysis. Differential expression was performed using limma/3.48.3. For the single-cell and single-nuclei RNAseq, reads were demultiplexed and aligned using cellranger/6.0.1. Then the samples were aggregated, normalized (SCTransform), and integrated (RunHarmony) using Seurat/4.1.0.

Public databases of human macrophages (GSE1925)^21^ and fibroblasts (GSE67737, GSE50954)^22,23^ treated with different types of interferon were used to explore the effects of such treatment in the expression of complement.

For visualization purposes, we used both the R and Python programming languages. Graphical analysis of single cell and single nuclei RNAseq data used the functions contained in Seurat/4.1.0. The Benjamini–Hochberg correction was used to adjust for multiple comparisons, and a corrected value of *p* (q value) ≤ 0.05 was considered statistically significant.

## Results

### Differential expression of complement proteins in the different types of IM

To define the transcriptomic profiles of patients with different types of IM, we performed bulk RNAseq on muscle biopsies from 132 IM patients, including those with DM (n = 44), AS (n = 18), IMNM (n = 54), and IBM (n = 16). The DM group included patients with autoantibodies recognizing Mi2 (n = 12), NXP2 (n = 14), TIF1*γ* (n = 12), and MDA5 (n = 6), whereas IMNM group consisted of patients with anti-HMGCR (n = 44) and anti-SRP (n = 10) autoantibodies. Thirty-three muscle biopsies from healthy comparators also underwent bulk RNAseq profling.

**Figure 1:**
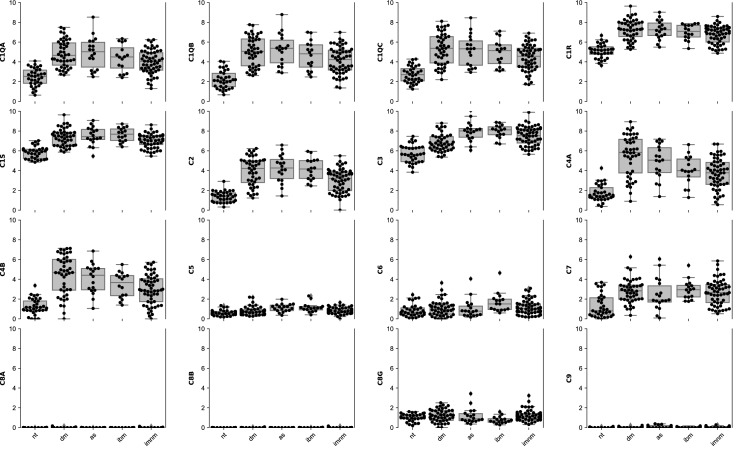
Expression of complement genes (log2[TMM + 1]) in normal muscle and in different types of inflammatory myopathy. The initial components of the complement cascade, C1–C4, were expressed at the highest levels in each type of myositis. C7 was expressed at an intermediate level, whereas C5, C6, and C8G were expressed at relatively low levels in myositis muscles. Genes encoding C5, C6, C8a, C8b, and C9 were expressed at very low or undetectable levels. Compared with other types of IM, biopsies from immune-mediated necrotizing myopathy patients had lower local levels of complement expression. Graphs were scaled to maximum value of all genes. nt, normal tissue; dm, dermatomyositis; as, antisynthetase syndrome; imnm, immune-mediated necrotizing myositis; ibm, inclusion body myositis.

Analysis of the bulk transcriptomic data revealed that the main complement components C1–C4 and C7 were overexpressed in muscle biopsies from patients with each type of IM compared with healthy comparator tissue (Fig. 1, Supplementary Fig. 1, Table 1). In each type of IM, the genes encoding C8a, C8b, and C9 were expressed at low or undetectable levels and were not differentially expressed compared to normal muscle (Fig. 1, Supplementary Fig. 1).

**Table 1:** Expression of complement genes in the different types of inflammatory myopathy compared to normal muscle.

Gene	DM		AS		IMNM		IBM	
	log2FC	q-value	log2FC	q-value	log2FC	q-value	log2FC	q-value
C1QA	2.7	2e−08	2.7	1e−07	1.8	1e−06	2.2	2e−06
C1QB	3.3	7e−09	3.4	2e−09	2.2	3e−07	2.8	1e−07
C1QC	2.8	7e−08	2.6	6e−08	1.9	3e−07	2.4	1e−07
C1R	2.2	4e−13	2.2	2e−10	1.7	1e−11	2.0	5e−09
C1S	1.6	4e−11	1.7	6e−09	1.2	3e−11	1.9	1e−09
C2	3.3	2e−08	3.5	6e−10	2.3	7e−08	3.5	5e−11
C3	1.3	3e−06	2.3	3e−09	1.8	1e−11	2.3	8e−09
C4A	4.4	7e−08	3.7	5e−09	2.5	4e−06	2.8	5e−07
C4B	4.4	3e−07	3.8	5e−08	2.6	3e−05	3.1	9e−07
C5	0.4	0.2	1.1	1e−04	0.6	0.002	1.2	3e−05
C6	0.3	0.5	0.3	0.5	0.7	0.08	1.7	3e−04
C7	2.7	2e−06	1.9	0.009	2.3	6e−05	2.7	6e−06
C8G	0.4	0.2	0.2	0.6	0.3	0.1	− 0.4	0.2

These genes did not pass the cutoff for differential expression: C8A, C8B, C9. *DM* dermatomyositis; *AS* Antisynthetase syndrome; *IMNM* Immune-mediated necrotizing myositis; *IBM* Inclusion body myositis.

Different types of IM had distinct complement gene expression profiles. For example, C6 was only overexpressed in muscle biopsies from IBM patients whereas C5 was overexpressed in muscle biopsies from patients with AS, IMNM, and IBM but not DM (Fig. 1, Supplementary Fig. 1). IMNM muscle biopsies had lower expression of C1–C2, and C4 compared to muscle biopsies from patients with other types of IM. In contrast, DM muscle biopsies were notable for increased expression of C4 and decreased expression of C3 and C5 relative to the other types of IM. Finally, IBM muscle biopsies were characterized by increased expression of C5 and C6 (Fig. 1, Supplementary Fig. 1, Table 2). Several complement activator and regulator genes were also deferentially expressed in the different types of IM (Supplementary Tables 1–4).

**Table 2:** Expression of complement genes in each group compared to the other types of inflammatory myopathy.

Gene	DM		AS		IMNM		IBM	
	log2FC	q-value	log2FC	q-value	log2FC	q-value	log2FC	q-value
C1QA	0.6	0.1	0.5	0.5	− 0.7	0.04	− 0.1	0.9
C1QB	0.6	0.1	0.7	0.4	− 0.9	0.02	0.0	1
C1QC	0.6	0.1	0.4	0.7	− 0.7	0.06	0.0	1
C1R	0.3	0.2	0.3	0.7	− 0.5	0.05	0.0	1
C1S	0.1	0.7	0.3	0.6	− 0.4	0.03	0.4	0.2
C2	0.5	0.2	0.7	0.4	− 1.0	0.005	0.6	0.3
C3	− 0.8	6e−04	0.6	0.2	0.1	0.8	0.6	0.1
C4A	1.5	7e−04	0.4	0.8	− 1.4	0.005	− 0.6	0.6
C4B	1.4	0.002	0.4	0.8	− 1.4	0.006	− 0.4	0.7
C5	− 0.5	0.007	0.6	0.2	0.0	0.9	0.6	0.04
C6	− 0.5	0.3	− 0.3	0.8	0.1	0.9	1.2	0.03
C7	0.3	0.5	− 0.6	0.6	− 0.1	0.8	0.3	0.7
C8G	0.2	0.4	0.0	1	0.2	0.6	− 0.8	0.06

These genes did not pass the cutoff for differential expression: C8A, C8B, C9. *DM* Dermatomyositis; *AS* Antisynthetase syndrome; *IMNM* Immune-mediated necrotizing myositis; *IBM* Inclusion body myositis.

### Correlation of complement expression with myositis activity

**Figure 2:**
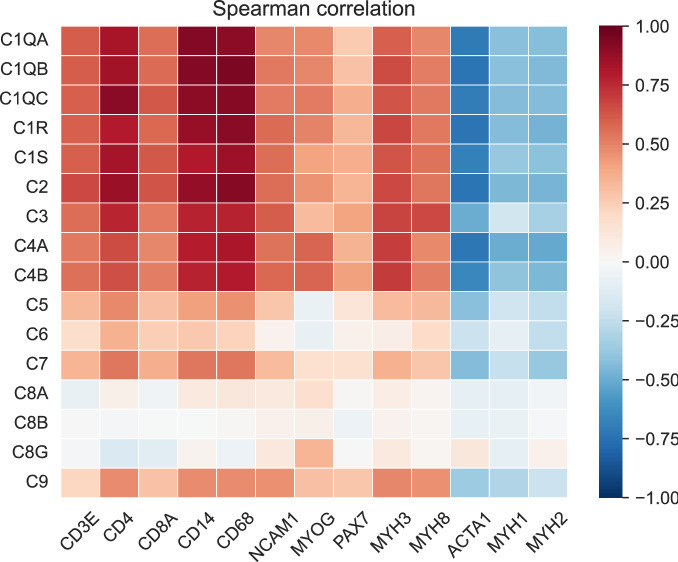
Correlation of complement expression with myositis disease activity. The analysis of bulk transcriptomic data showed that the local expression of complement was positively correlated with markers of muscle regeneration (NCAM1, MYOG, PAX7, MYH3, and MYH8) and canonical T-cell and monocyte/macrophage markers (CD3E, CD4, CD8A, CD14, and CD68). In contrast, there was a negative correlation between the expression of complement proteins with markers of mature muscle cells markers (ACTA1, MYH1, and MYH2).

Next, we analyzed the bulk transcriptomic data to determine whether complement expression levels were correlated with the degree of myositis disease activity. As shown in Fig. 2, the local expression of complement was positively correlated with markers of muscle regeneration (NCAM1, MYOG, PAX7, MYH3, and MYH8) and canonical T-cell and monocyte/macrophage markers (CD3E, CD4, CD8A, CD14, and CD68). In contrast, there was a negative correlation between the expression of complement proteins with markers of mature muscle cells (ACTA1, MYH1, and MYH2) (Fig. 2).

Of note, two muscle biopsies were available from the same anti-Mi2-positive DM patient. The first biopsy was obtained when the patient had very active muscle disease and the second biopsy was obtained when the patient had minimal myositis activity, 5 months after starting a JAK/STAT inhibitor (i.e., tofacitinib). Consistent with the cross-sectional data, the expression of complement genes markedly declined as the patient’s myositis became less active (Supplementary Fig. 2).

### Different cell types coordinately express various complement genes

**Figure 3:**
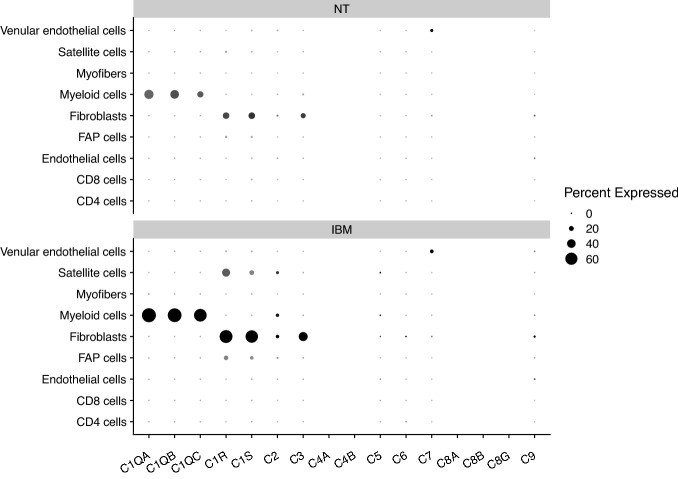
Single-cell RNA sequencing analysis of complement genes from fresh muscle tissue. Biopsies from 3 patients with suspected IBM and 3 healthy volunteers were included. C1QA, C1QB, and C1QC were expressed at the highest levels in CD14 + /CD68 + myeloid cells (i.e., macrophages) whereas genes encoding C1R, C1s, and C3 were primarily expressed in fibroblasts. Unlike in healthy muscle, IBM muscle biopsies showed expression of C1R, and C1S in satellite cells and, to a lesser degree, in FAP cells. NT, Normal tissue; IBM, Inclusion body myositis; FAP, Fibroadipogenic progenitors.

Muscle tissue includes many different cell types. To determine which cell types express complement genes, we performed single-cell RNAseq on fresh muscle tissue derived from 3 patients undergoing a muscle biopsy for suspected IBM and three healthy volunteers. As shown in Supplementary Fig. 3, cell clusters representing myofibers, satellite cells, myeloid cells, venular endothelial cells, endothelial cells, fibroblasts, fibroadipogenic progenitors (FAP), CD4+ T cells, and CD8+ T cells could be identified. Genes encoding C1QA, C1QB, and C1QC were expressed at the highest levels in CD14+/CD68+ myeloid cells (i.e., macrophages) whereas genes encoding C1R, C1S, and C3 were primarily expressed in fibroblasts (Fig. 3, Supplementary Fig. 4). Unlike in healthy muscle, suspected IBM muscle biopsies showed expression of C1R, and C1S in satellite cells and, to a lesser degree, in FAP cells.

To validate the gene expression data from the single-cell experiments, we performed single-nuclei RNAseq using a subset of 15 frozen muscle biopsy specimens. This included biopsies from 4 patients with DM (2 with anti-Mi2 and 2 with anti-NXP2 autoantibodies), 3 patients with anti-Jo1-positive AS, 6 patients with IMNM (4 with anti-HMGCR and 2 with anti-SRP autoantibodies), and 2 patients with IBM. As shown in Supplementary Fig. 5, transcriptomic data from single-nuclei could be used to identify clusters of cells representing mature muscle fibers, satellite cells (i.e., muscle cell precursors), endothelial cells, fibroblasts, T cells, myeloid cells, FAP cells, and adipocytes.

Confirming the results of the single-cell experiment, genes encoding C1QA, C1QB, and C1QC were primarily expressed by myeloid cells (Supplementary Figs. 6,7). In contrast, the genes encoding C1R, C1S, and C3 were predominantly expressed by fibroblasts. C1R and C1S were expressed by satellite cells and FAP, albeit at lower levels than in fibroblasts (Supplementary Figs. 6,7).

### Local complement expression in IM correlates with IFN*γ* pathway activation

**Figure 4:**
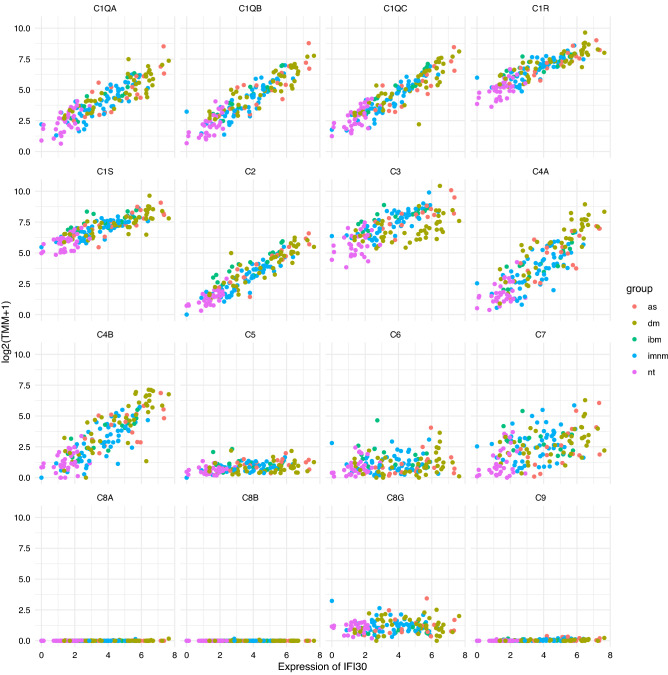
Correlation of IFI30 with complement genes in normal muscle and different types of inflammatory myopathy. The expression of IFI30, an IFNg-stimulated gene, strongly correlates with the expression of the initial components of the complement cascade. nt, normal tissue; dm, dermatomyositis; as, antisynthetase syndrome; imnm, immune-mediated necrotizing myositis; ibm, inclusion body myositis.

As IFN*γ* is known to induce the expression of several complement genes in cultured muscle cells, macrophages, and fibroblasts^24–27^, and IFN*γ*-stimulated genes are expressed at high levels in certain types of IM^17^, we studied the association between IFN*γ*-stimulated gene and complement gene expression in our bulk transcriptomic data. This analysis revealed a strong correlation between the expression of prominent IFN*γ*-stimulated genes (e.g., IFI30, and GBP2) and complement genes (Fig. 4, Supplementary Fig. 8).

Next, we sought to determine whether IFN*γ* or other interferons could stimulate the expression of complement genes in the complement-expressing cells identified in the single-nuclei and single-cell RNAseq experiments.

**Figure 5:**
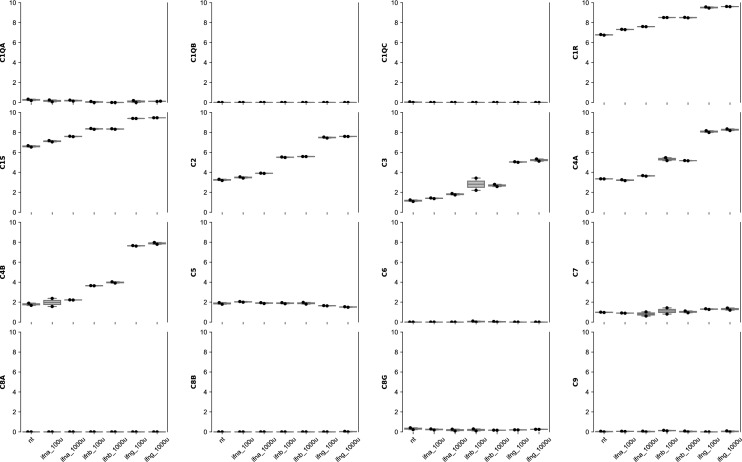
Effect of different types of interferon on complement genes in differentiating human skeletal muscle myoblasts. Increase of the expression (log2[TMM + 1]) of C1R, C1S, and C2–C4 with IFNg. Scaled to the maximum value of all genes. nt, untreated; ifna_100u: treated with 100U of IFNA; ifna_1000u: treated with 1000U of IFNA; ifnb_100u: treated with 100U of IFNB1; ifnb_1000u: treated with 1000U of IFNB1; ifng_100u: treated with 100U of IFNG;. ifng_1000u: treated with 1000U of IFNG.

In cultured human skeletal muscle cells, C1R, C1S, C2–C5, and C7, were expressed during the differentiation of myoblasts into myotubes, with the largest increase occurring within the first two days of differentiation (Supplementary Fig. 9). Furthermore, treatment of human myoblasts with IFN*γ* markedly increased the baseline expression of C1R, C1S, and C2–C4. Treatment of cultured muscle cells with IFN$\beta 1$ also induced the expression of some complement genes, albeit to a lesser degree. However, treatment of cultured muscle cells with IFN$\alpha$ had little effect on the expression of complement genes (Fig. 5). Taken together, these results support the hypothesis that both muscle differentiation and IFN*γ* stimulate complement gene expression in human myoblasts and myotubes.

We utilized publicly available datasets to determine whether IFN*γ* modulates complement gene expression in cultured human macrophages and fibroblasts. Indeed, human macrophages treated with IFN*γ* showed a marked increase in the expression of C1R, C1S, C2, and especially C1QB (Supplementary Fig. 10). Similarly, fibroblasts express C1R and C1S at high levels and treatment with IFN*γ* results in a small, but consistent, increase in the expression of these two genes (Supplementary Figs. 11,12).

## Discussion

In this study, we used bulk transcriptomic data from human muscle biopsies to demonstrate that various complement genes are expressed locally within muscle tissue and that local complement expression levels correlate with IM disease activity. The bulk transcriptomic data also revealed that muscle from each type of IM has a distinct “complement expression signature”. We then used single-nuclei and single-cell RNAseq techniques to show that macrophages, fibroblasts, and satellite cells are the primary cell types within the muscle that express complement genes. Moreover, we showed that macrophages predominantly express certain complement genes (i.e., C1QA, C1QB, and C1QC) whereas fibroblasts (i.e., C1R, C1S, and C3), satellite cells (i.e., C1R, and C1S), and, to a lower extent, fibroadipocytes (i.e., C1R, and C1S), express a different set of complement genes. Finally, we showed that the expression of complement genes is highly correlated with the expression of IFN*γ*-stimulated genes in IM muscle and that IFN*γ* induces complement overexpression in differentiating human skeletal muscle myoblasts, macrophages, and, at least to some degree, in fibroblasts. This suggests that the overexpression of complement genes in patients with myositis is not only due to an increased number of cells expressing complement, but also the result of a more intense expression in each of those cells induced by IFN*γ*. In this regard, it’s worth noting that IMNM has the lowest expression levels of both IFN*γ*-stimulated genes and complement genes.

The pathophysiologic relevance of local complement production has been studied in other inflammatory diseases. For example, C3 expression by synovial fibroblasts has been linked to inflammation-mediated tissue priming in arthritis^28^. Furthermore, analogous to what we have shown here for IM muscle, different complement genes are differentially expressed by different cells of the lung^29^. Specifically, lung macrophages express C1QA, C1QB, and C1QC, whereas lung mesothelial cells and fibroblasts expressed C1R, C1S, and C3^29^. Taken together, these findings suggest that the coordinated local expression of complement genes is not specific to muscle tissue in IM but, rather, may be a general mechanism to regulate the complement pathway in inflamed tissues.

This paper has several limitations. First, the expression of certain complement genes fell below the detection threshold of the sequencing techniques that we used. For example, C8a and C8b could not be detected in the bulk RNAseq. Moreover, several genes, including C4a and C4b, were detected by bulk RNAseq but could not be identified by single-nuclei or single-cell RNAseq. Second, we used only RNA-based sequencing methods to study the local expression of complement, because it is selective for locally-synthesized complement genes. While this has numerous advantages, including the application of single-nuclei and single-cell sequencing technologies, we cannot estimate the efficiency of the local translation of complement genes from RNA to protein. Finally, we restricted our analysis to the most common types of IM and the less common types of IM may have different patterns of complement expression.

In summary, this transcriptomic analysis has revealed that macrophages, fibroblasts, and satellite cells express complement genes in a complex and highly coordinated manner within IM muscle biopsies. We also provide evidence that the local expression of complement genes may be regulated by IFN*γ*, a cytokine already known to be a key player in IM pathogenesis. Future studies will be required for a more complete understanding of the pathophysiologic role of the complement system in myositis muscle and other inflamed tissues.

## Supplementary Information

Supplementary Information.

## Data Availability

The datasets generated and/or analysed during the current study are available in the Gene Expression Omnibus repository (GSE220915).
