# Supplementary material for: Coordinated local RNA overexpression of complement induced by interferon gamma in myositis
Source: Sci Rep. 2023 Feb 4;13:2038. doi: 10.1038/s41598-023-28838-z (PMC9899209; doi:10.1038/s41598-023-28838-z)
Supplement: Supplementary file 1 — Supplementary Information. [file 41598_2023_28838_MOESM1_ESM.pdf]

Supplementary Figure 1. Expression of complement genes (log2[TMM+1]) in normal muscle and in different types of inflammatory myopathy. The initial components of the complement cascade, C1-C4, were expressed at the highest levels in each type of myositis. C7 was expressed at an intermediate level, whereas C5, C6, and C8G were expressed at relatively low levels in myositis muscles. Genes encoding C5, C6, C8a, C8b, and C9 were expressed at very low or undetectable levels. Compared with other types of IM, biopsies from immune-mediated necrotizing myopathy patients had lower local levels of complement expression. Scaled to maximum value of each gene.

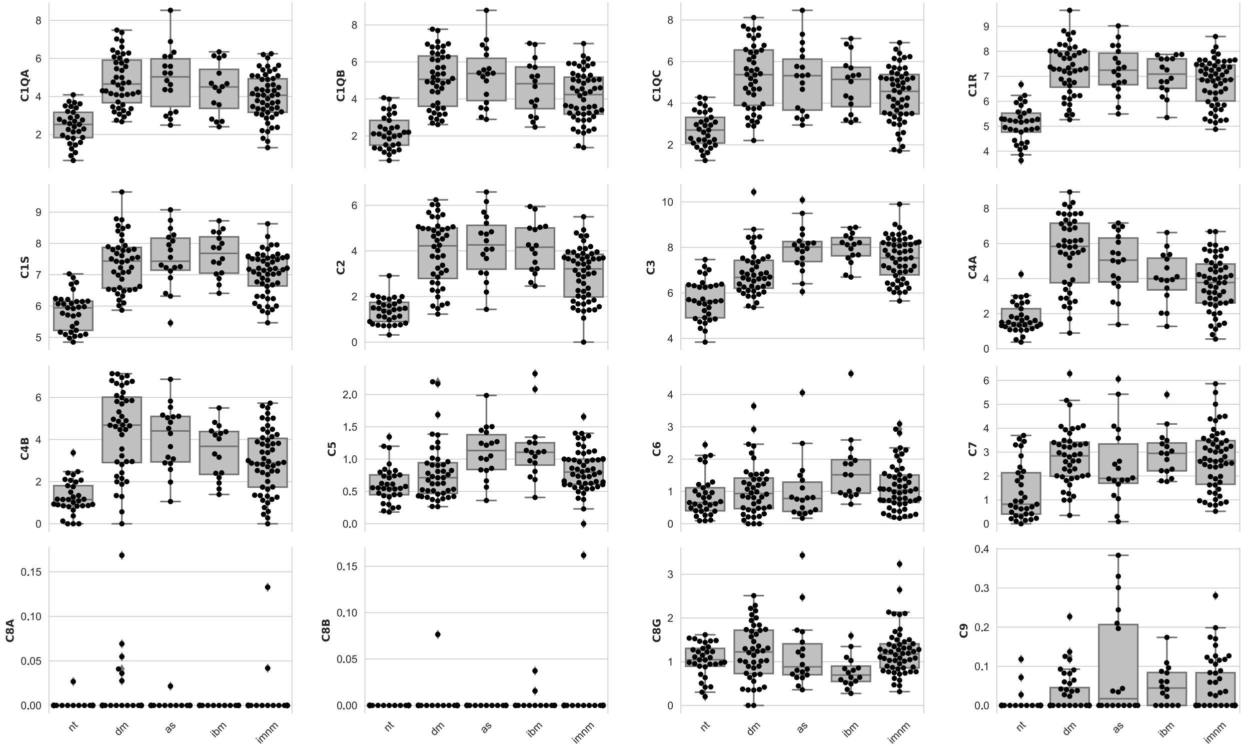

nt: normal tissue; dm: dermatomyositis; as: antisynthetase syndrome; imnm: immune-mediated necrotizing myositis; ibm: inclusion body myositis.

Supplementary Table 1. Expression of complement activator genes in the different types of inflammatory myopathy compared to normal muscle.

Gene	DM		AS		IMNM		IBM	
	log2FC	q-value	log2FC	q-value	log2FC	q-value	log2FC	q-value
**CFB**	3.9	9e-09	3.1	1e-10	2.0	4e-09	2.5	1e-09
**CFD**	0.5	0.07	0.0	1	0.4	0.05	0.8	0.007
**CFP**	1.7	7e-06	1.7	4e-05	1.3	6e-05	1.7	2e-05
**FCN1**	1.8	5e-04	1.9	5e-05	1.6	2e-04	1.9	8e-04
**FCN3**	1.7	1e-04	0.2	0.7	0.6	0.2	-0.3	0.5
**MASP1**	-1.6	4e-06	-2.2	3e-05	-1.0	0.001	-0.4	0.4

These genes did not pass the cutoff for differential expression: COLEC10, COLEC11, FCN2, MASP2, MBL2. *DM: dermatomyositis; AS: antisynthetase syndrome; IMNM: immune-mediated necrotizing myositis; IBM: inclusion body myositis*

Supplementary Table 2. Expression of complement activator genes in each group compared to the other types of inflammatory myopathy.

Gene	DM		AS		IMNM		IBM	
	log2FC	q-value	log2FC	q-value	log2FC	q-value	log2FC	q-value
**CFB**	1.5	2e-06	0.3	0.8	-1.4	7e-05	-0.4	0.6
**CFD**	0.1	0.9	-0.5	0.4	0.0	1	0.5	0.4
**CFP**	0.2	0.5	0.3	0.7	-0.4	0.2	0.2	0.7
**COLEC11**	0.7	0.1	0.2	0.9	-0.5	0.3	-0.5	0.5
**FCN1**	0.1	0.9	0.3	0.8	-0.2	0.7	0.1	0.9
**FCN3**	1.3	1e-04	-0.6	0.6	-0.4	0.4	-1.3	0.07
**MASP1**	-0.6	0.1	-0.9	0.3	0.5	0.2	1.1	0.04

These genes did not pass the cutoff for differential expression: COLEC10, FCN2, MASP2, MBL2. *DM: dermatomyositis; AS: antisynthetase syndrome; IMNM: immune-mediated necrotizing myositis; IBM: inclusion body myositis*

Supplementary Table 3. Expression of complement regulator genes in the different types of inflammatory myopathy compared to normal muscle.

Gene	DM		AS		IMNM		IBM	
	log2FC	q-value	log2FC	q-value	log2FC	q-value	log2FC	q-value
**C3AR1**	2.1	3e-06	2.5	4e-07	2.0	3e-06	2.3	9e-07
**C5AR1**	2.2	2e-07	2.3	9e-08	1.9	8e-08	1.6	3e-07
**CD46**	-0.5	8e-04	-0.4	0.001	-0.4	2e-06	0.1	0.4
**CD55**	-0.2	0.3	-0.2	0.08	-0.1	0.5	-0.3	0.05
**CD59**	-0.3	0.002	-0.5	5e-05	-0.2	0.005	-0.2	0.07
**CD93**	-0.3	0.07	0.1	0.4	0.0	0.7	0.2	0.1
**CFH**	1.3	2e-06	1.8	2e-07	1.4	6e-09	1.8	4e-08
**CFHR1**	3.3	3e-04	4.4	8e-06	2.9	0.005	1.9	0.02
**CFHR3**	1.2	0.03	2.3	5e-05	1.0	0.04	1.9	6e-04
**CFI**	0.9	0.002	1.1	3e-04	0.8	5e-05	1.1	4e-07
**CLU**	1.2	8e-06	1.1	0.003	1.0	2e-04	1.4	2e-04
**CR1**	0.9	0.08	2.8	6e-09	2.0	2e-05	2.4	1e-05
**CSMD1**	1.0	0.01	1.0	0.06	1.8	3e-07	1.0	0.02
**ELANE**	0.6	0.07	-0.2	0.6	0.5	0.1	0.5	0.2
**ITGAM**	1.1	6e-04	1.8	2e-08	1.3	2e-07	1.9	2e-07
**ITGAX**	1.4	6e-04	1.8	2e-05	1.7	5e-07	2.0	7e-05
**ITGB2**	1.6	4e-05	2.4	8e-08	1.7	2e-07	2.3	8e-07
**SERPING1**	1.6	3e-11	1.3	8e-07	1.0	5e-09	1.4	5e-08
**VSIG4**	2.2	3e-07	2.7	6e-08	2.2	1e-07	2.2	3e-08
**VTN**	-0.3	0.5	-1.3	0.02	-0.3	0.4	-0.5	0.3

These genes did not pass the cutoff for differential expression: C4BPA, C4BPB, CFHR2, CFHR4, CFHR5, CR2, CSMD2, CSMD3, F2. *DM: dermatomyositis; AS: antisynthetase syndrome; IMNM: immune-mediated necrotizing myositis; IBM: inclusion body myositis*

Supplementary Table 4. Expression of complement regulator genes in each group compared to the other types of inflammatory myopathy.

Gene	DM		AS		IMNM		IBM	
	log2FC	q-value	log2FC	q-value	log2FC	q-value	log2FC	q-value
**C3AR1**	-0.1	0.7	0.5	0.5	-0.2	0.6	0.3	0.7
**C4BPB**	-1.0	0.02	1.4	0.03	0.1	0.9	0.5	0.5
**C5AR1**	0.2	0.6	0.4	0.5	-0.2	0.7	-0.5	0.4
**CD46**	-0.2	0.06	0.0	1	0.0	1	0.5	0.008
**CD55**	-0.1	0.6	-0.1	0.9	0.2	0.3	-0.1	0.6
**CD59**	-0.1	0.2	-0.2	0.4	0.2	0.09	0.1	0.6
**CD93**	-0.5	9e-04	0.3	0.5	0.2	0.3	0.3	0.3
**CFH**	-0.3	0.2	0.4	0.4	-0.1	0.8	0.4	0.3
**CFHR1**	0.3	0.7	1.6	0.2	-0.4	0.6	-1.4	0.3
**CFHR3**	-0.3	0.5	1.2	0.1	-0.6	0.2	0.7	0.3
**CFI**	-0.1	0.7	0.3	0.7	-0.1	0.7	0.2	0.6
**CLU**	0.1	0.7	0.0	1	-0.2	0.4	0.3	0.5
**CR1**	-1.4	2e-04	1.2	0.05	0.4	0.4	0.8	0.2
**CSMD1**	-0.6	0.07	-0.3	0.7	0.8	0.003	-0.3	0.6
**ELANE**	0.2	0.6	-0.6	0.5	0.1	0.9	0.0	1
**ITGAM**	-0.5	0.04	0.6	0.3	0.0	0.9	0.6	0.1
**ITGAX**	-0.5	0.2	0.3	0.8	0.1	0.8	0.4	0.5
**ITGB2**	-0.4	0.2	0.7	0.3	-0.2	0.6	0.6	0.2
**SERPING1**	0.4	0.03	0.0	1	-0.4	0.01	0.1	0.7
**VSIG4**	-0.2	0.6	0.6	0.4	-0.1	0.9	0.0	0.9
**VTN**	0.2	0.7	-0.9	0.3	0.3	0.5	-0.1	0.9

These genes did not pass the cutoff for differential expression: C4BPA, CFHR2, CFHR4, CFHR5, CR2, CSMD2, CSMD3, F2. *DM: dermatomyositis; AS: antisynthetase syndrome; IMNM: immune-mediated necrotizing myositis; IBM: inclusion body myositis*

Supplementary Figure 2. Complement gene expression levels before and after treatment with tofacitinib (Tofa) in an anti-Mi2-positive dermatomyositis patient.

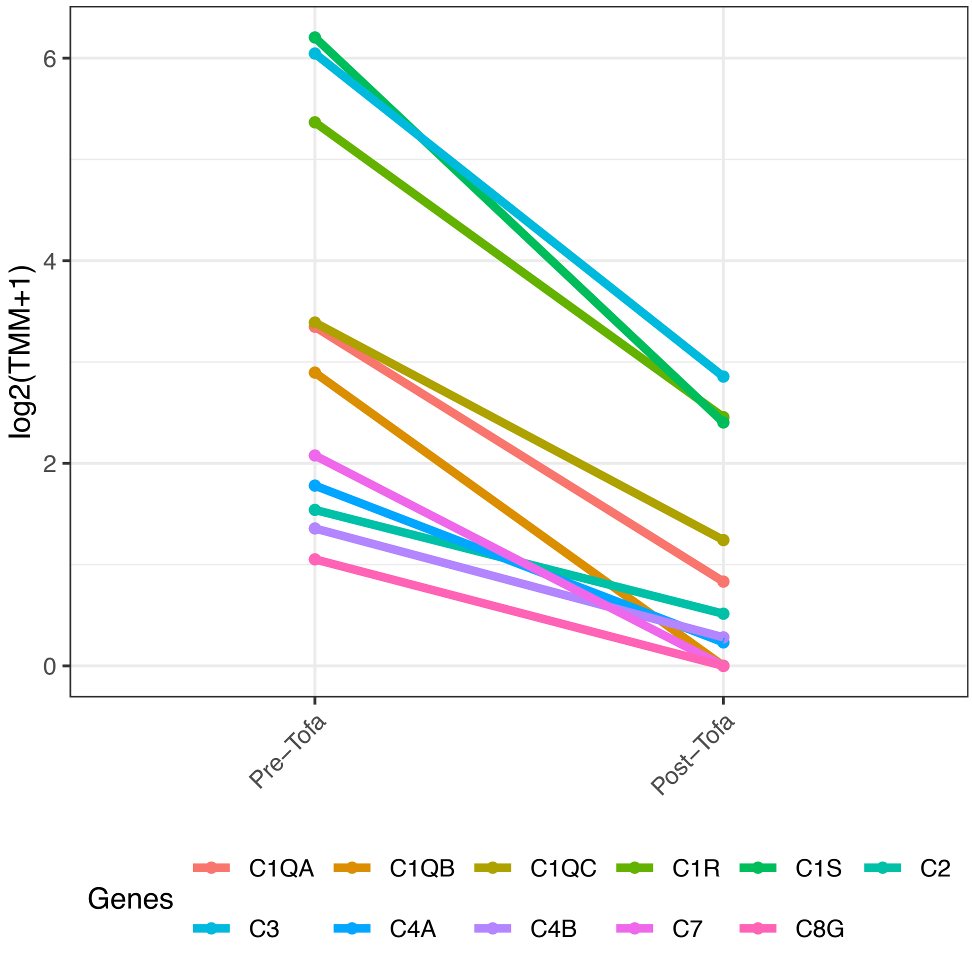

Supplementary Figure 3. Representative genes in each cluster by single-cell RNAseq.

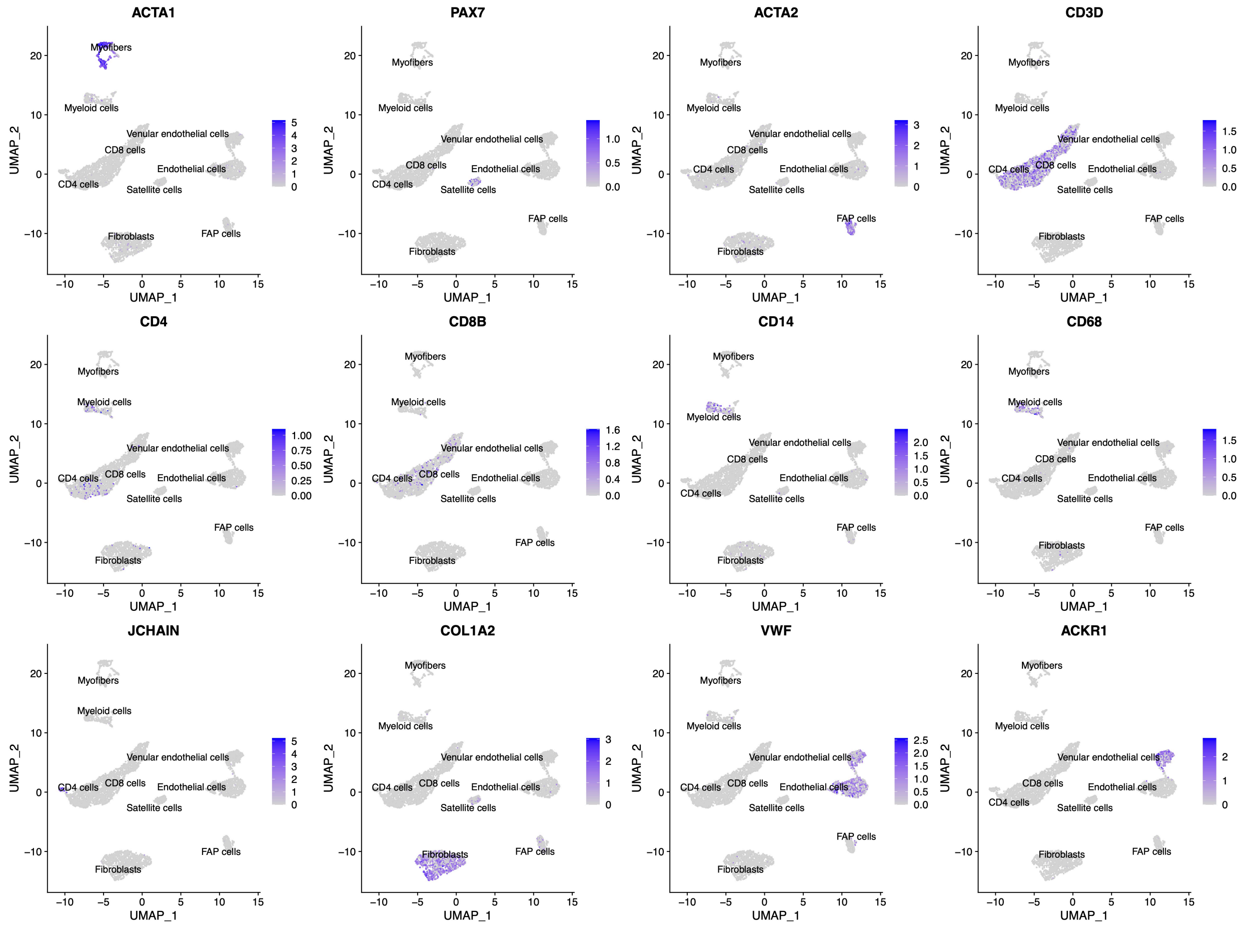

Supplementary Figure 4. Single-cell RNAseq analysis of the expression of complement genes in different muscle cells from fresh muscle tissue. Samples derived from biopsies of 3 patients with a suspected IBM and 3 healthy volunteers were included. Genes encoding C1QA, C1QB, and C1QC were expressed at the highest levels in CD14+/CD68+ myeloid cells (i.e., macrophages) whereas genes encoding C1r, C1s, and C3 were primarily expressed in fibroblasts.

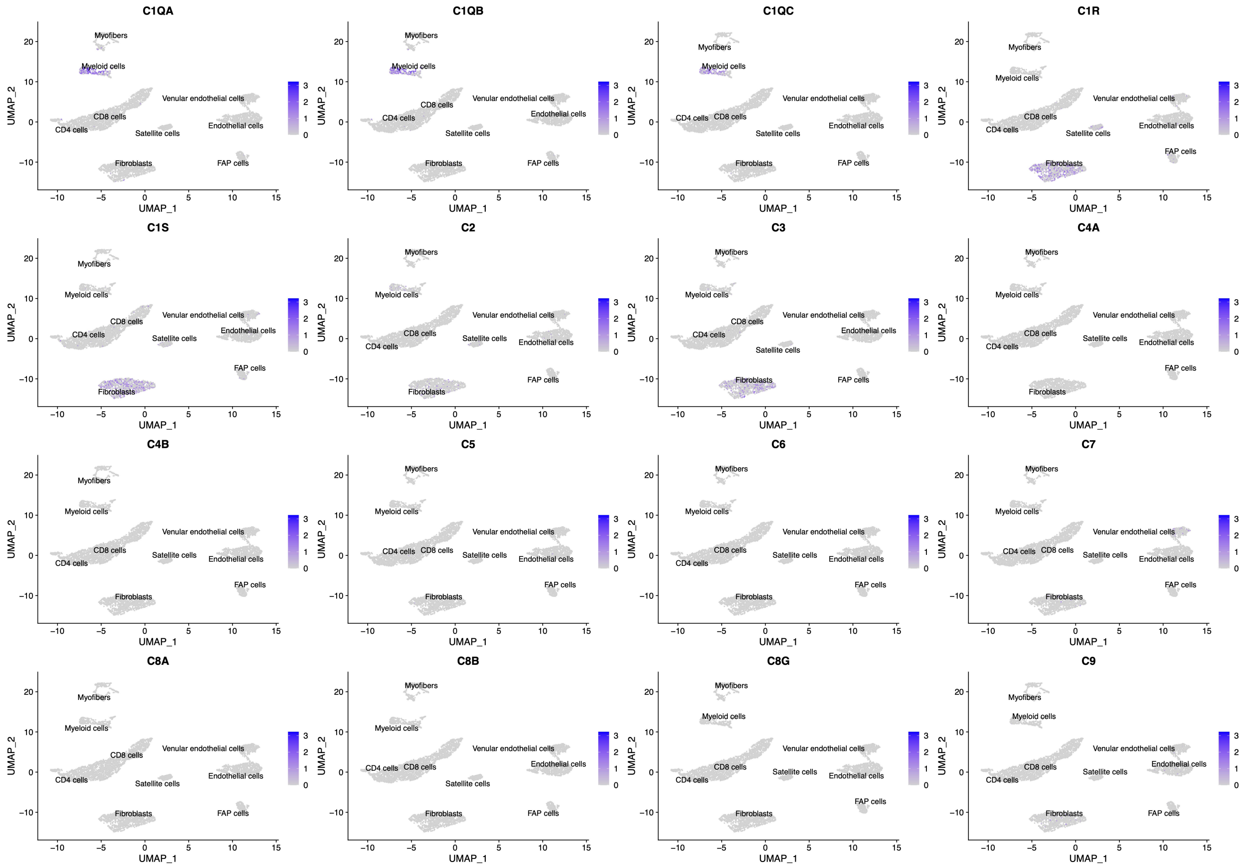

Supplementary Figure 5. Representative genes in each cluster by single-nuclei RNAseq.

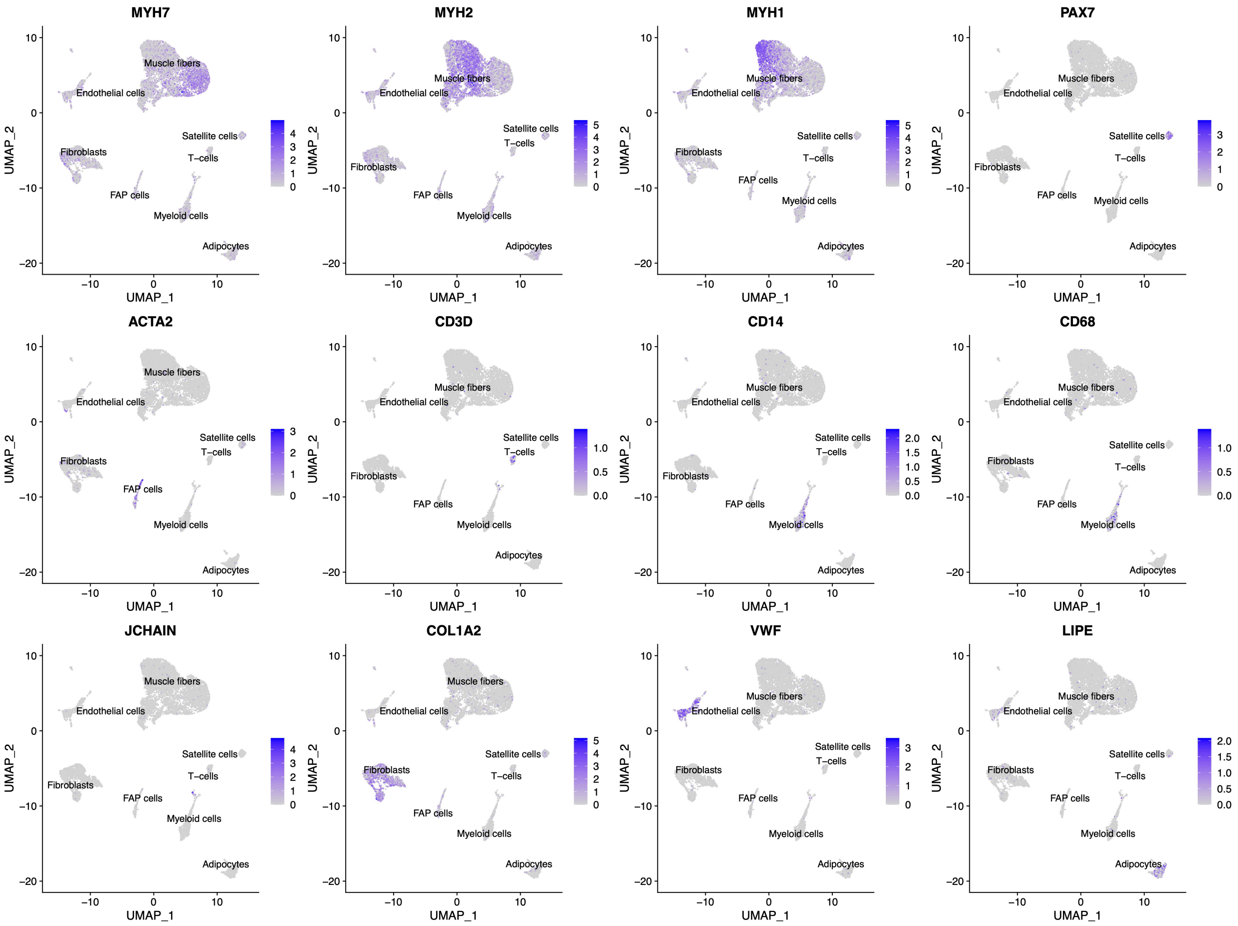

Supplementary Figure 6. Expression of complement genes in different muscle cells and different types of inflammatory myopathy by single-nuclei RNAseq.

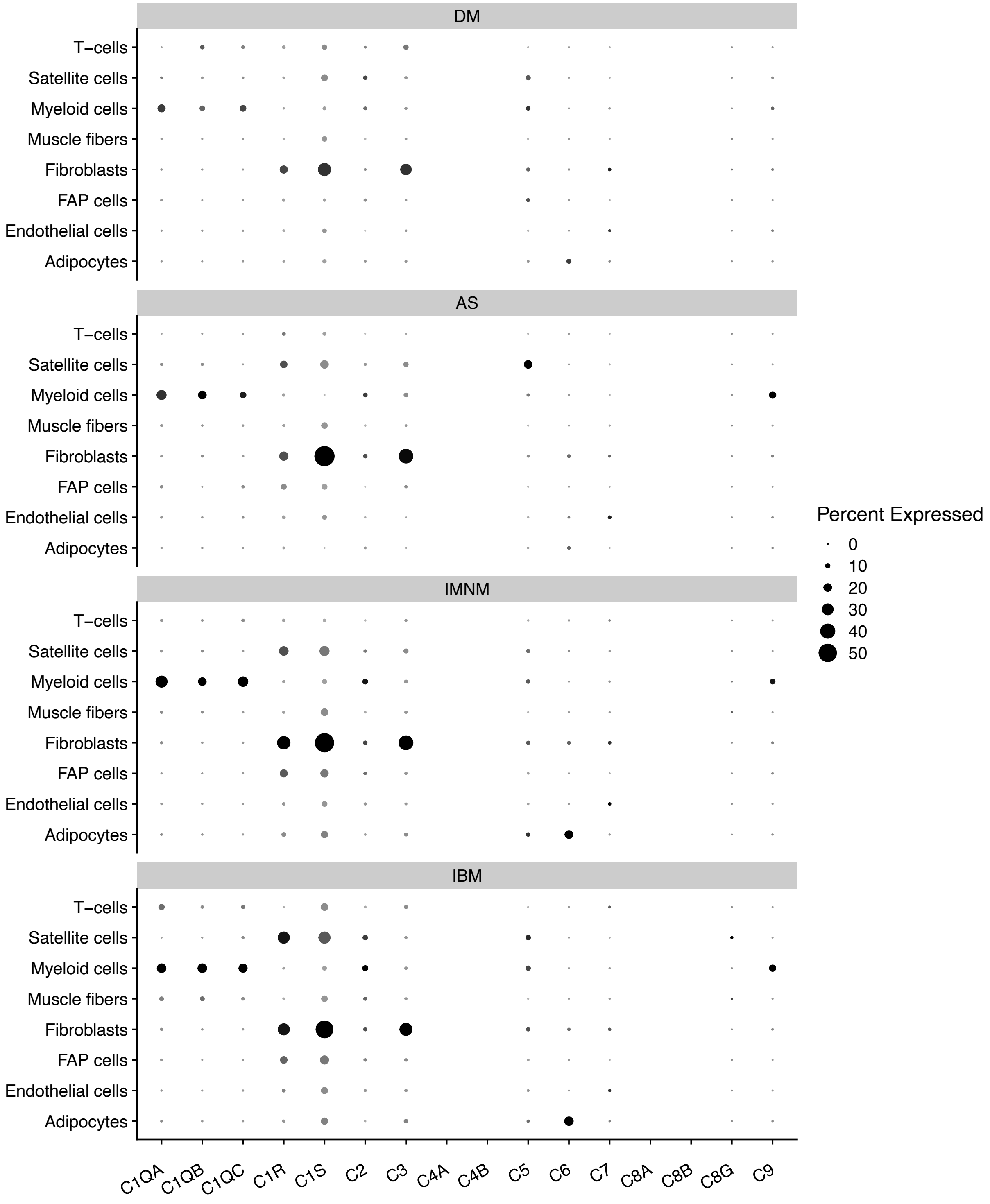

Supplementary Figure 7. Expression of complement genes in different muscle cells by single-nuclei RNAseq. Single nuclei RNAseq data form 15 frozen muscle biopsies (4 patients with DM, 3 patients with anti-Jo1-positive AS, 6 patients with IMNM, and 2 patients with IBM) was analyzed to determine which cell types express complement genes. Genes encoding C1qA, C1qB, and C1qC were primarily expressed by myeloid cells, whereas genes encoding C1R, C1s, and C3 were primarily expressed in fibroblasts.

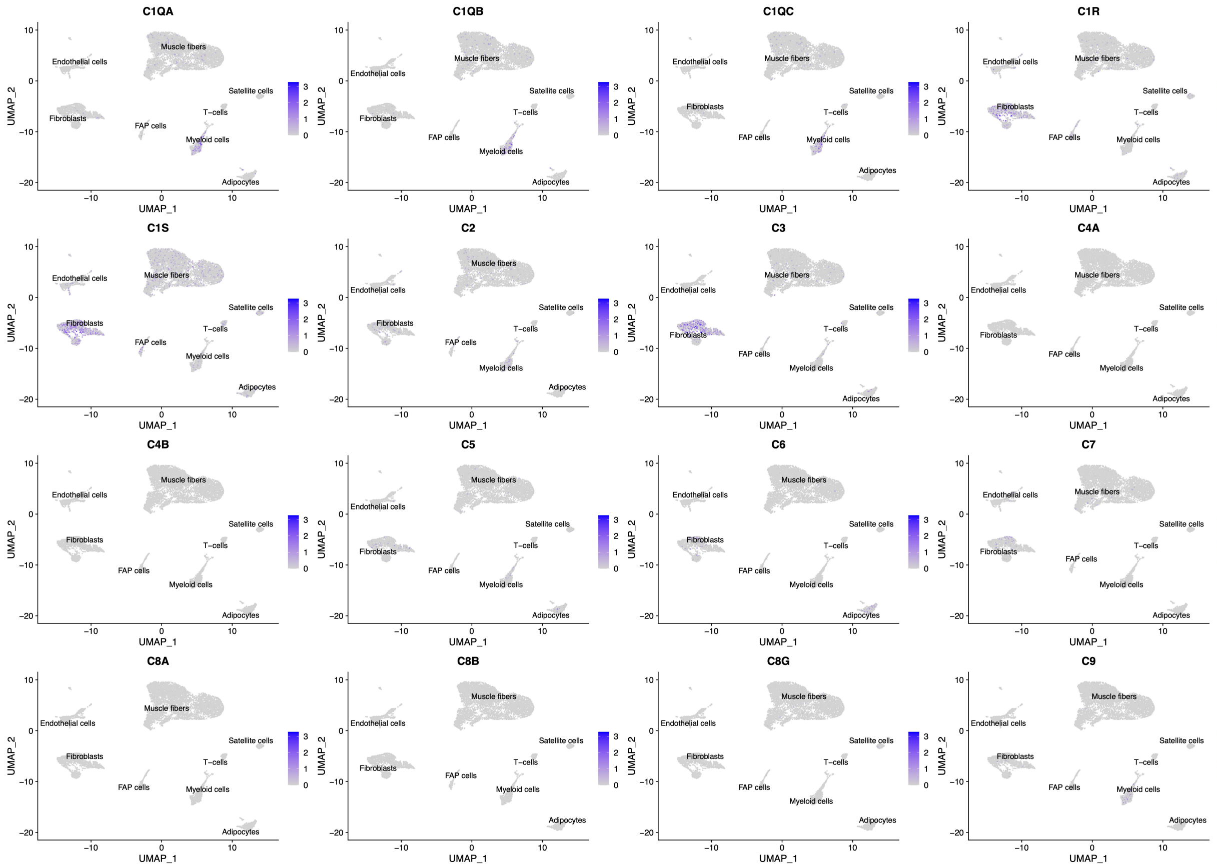

DM: dermatomyositis; AS: antisynthetase syndrome; IMNM: immune-mediated necrotizing myositis; IBM: inclusion body myositis.

Supplementary Figure 8. Correlation of GBP2 with complement genes in normal muscle and in different types of inflammatory myopathy. The expression of GBP2, an IFNg-stimulated gene, strongly correlates with the expression of the initial components of the complement cascade.

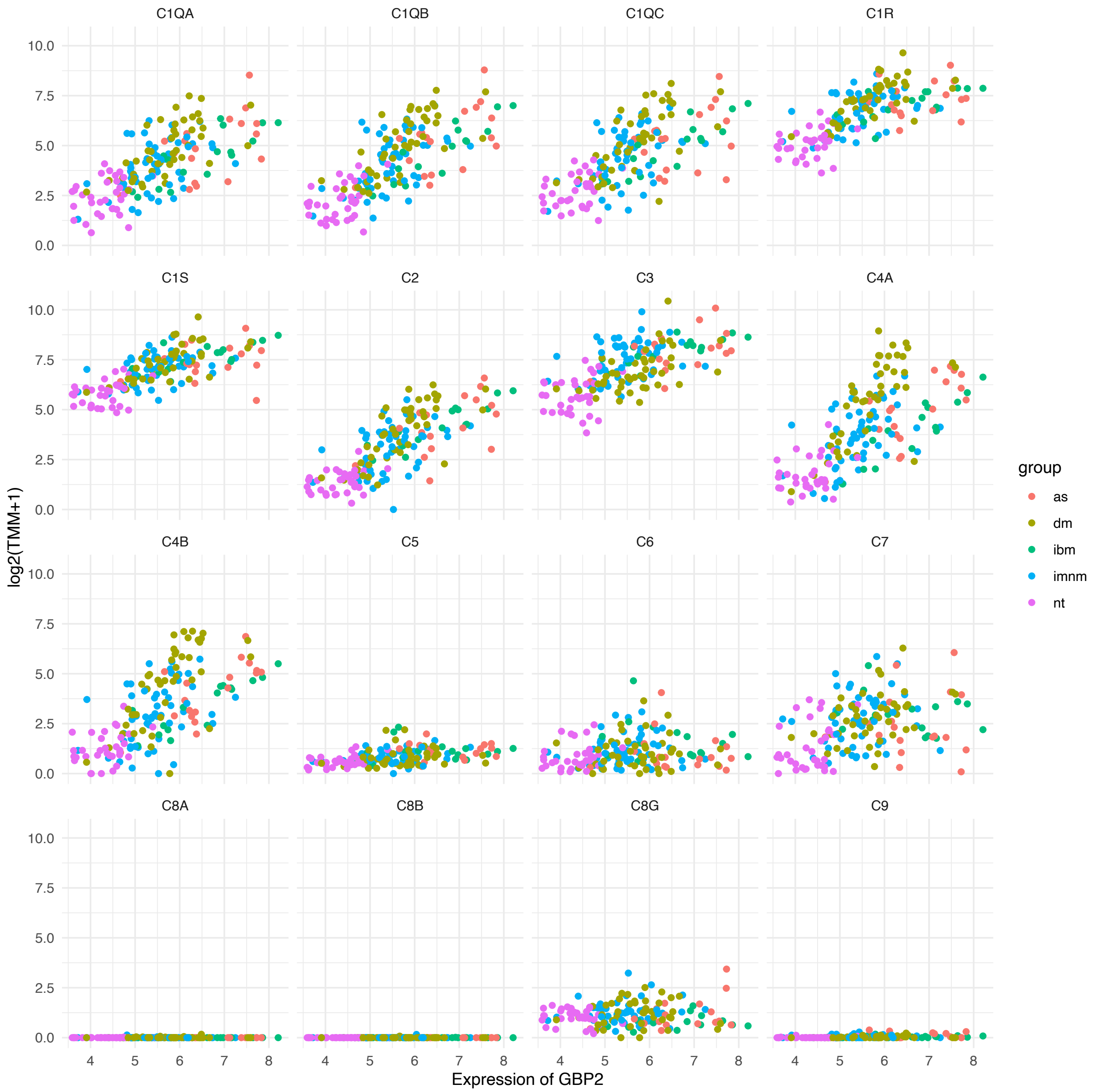

nt: normal tissue; dm: dermatomyositis; as: antisynthetase syndrome; imnm: immune-mediated necrotizing myositis; ibm: inclusion body myositis.

Supplementary Figure 9. Expression of complement genes (log2[TMM+1]) in differentiating human skeletal muscle myoblasts. Overexpression of various complement genes, most predominantly C1R and C1S, early after starting differentiation. Scaled to the maximum value of all genes.

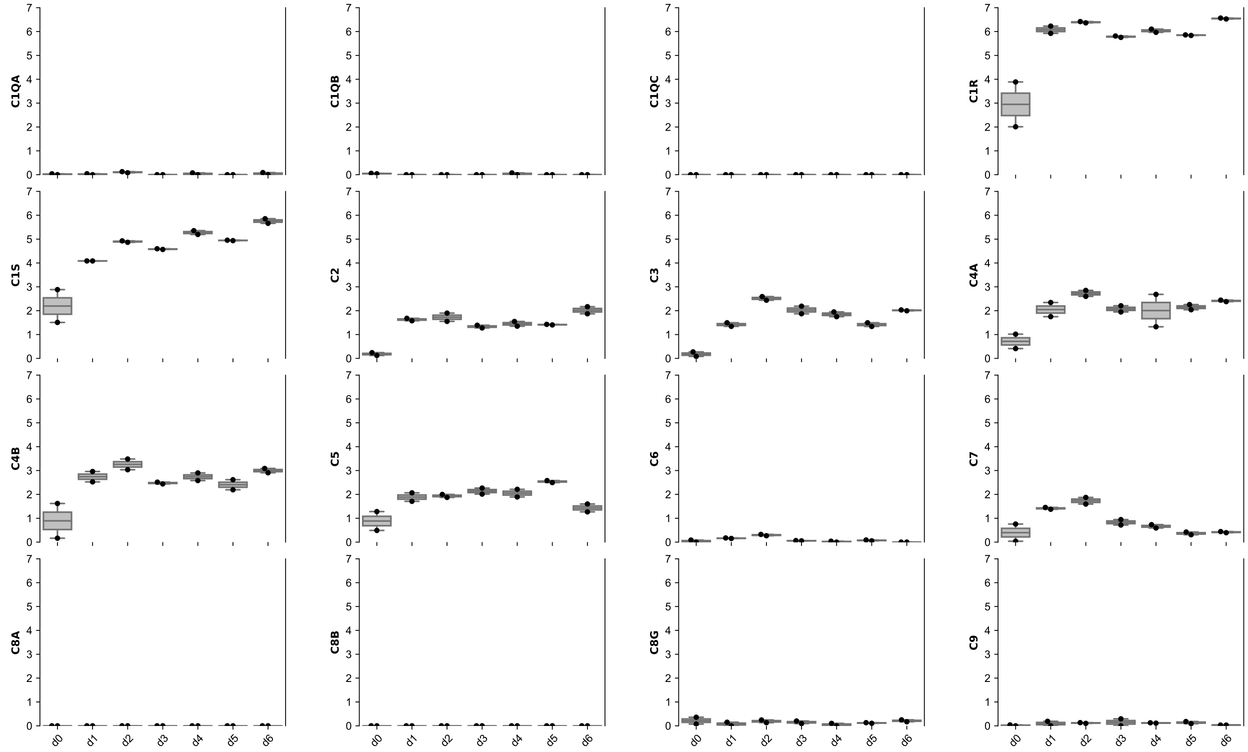

d0: before start of differentiation media; d1-d6: days after starting the differentiation media.

Supplementary Figure 10. Effect of interferon gamma on complement genes in macrophages from GSE1925. Macrophages treated with IFNg overexpressed the initial components of the complement pathway (C1QB, C1R, C1S, C2). C1QB had the highest detectable expression among complement genes.

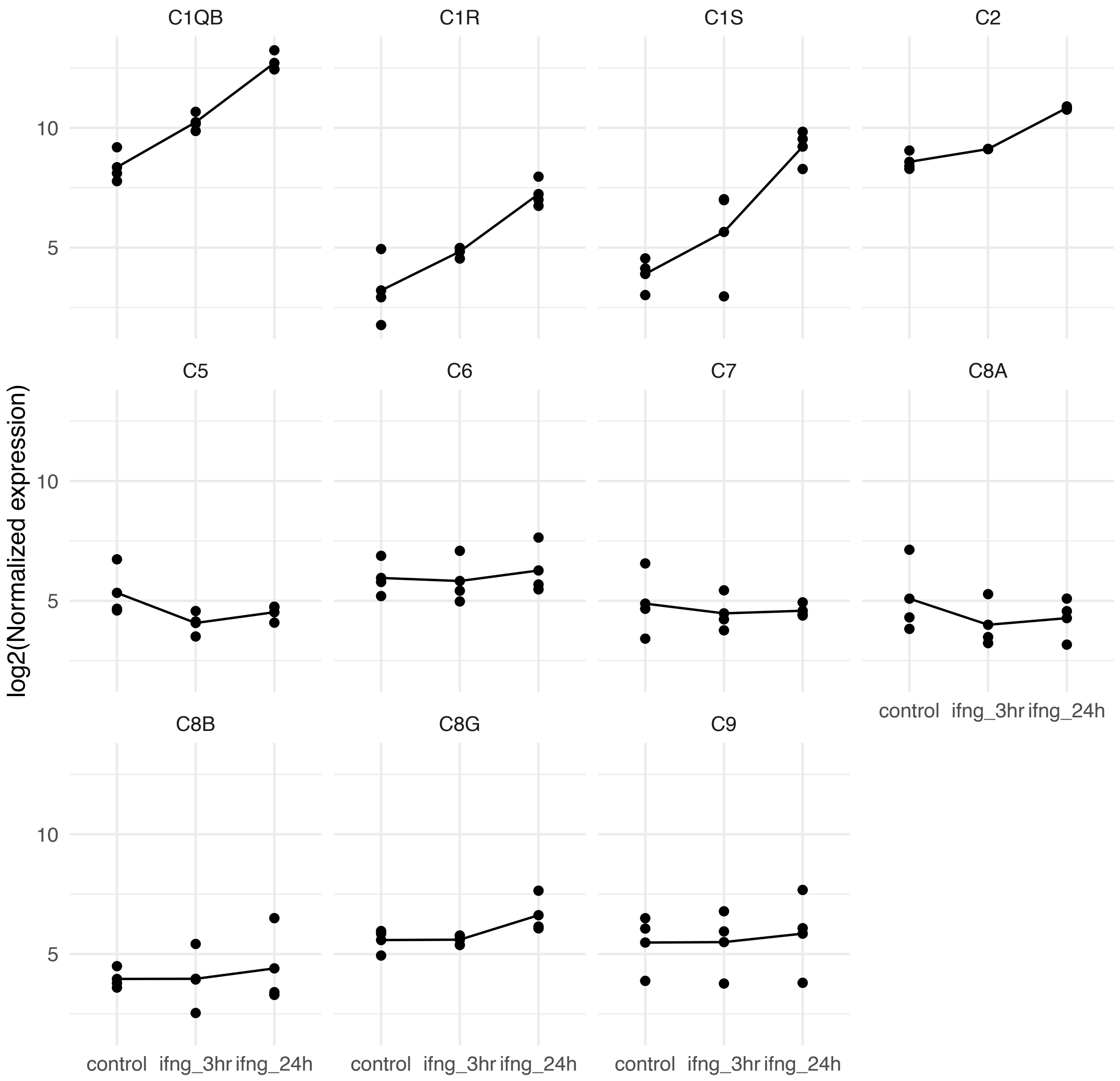

Scaled to the maximum value of all genes. control: untreated macrophages; ifng_3hr: treatment with 100 U/ml of interferon gamma for 3 hours: ifng_24h: treatment with 100 U/ml of interferon gamma for 24 hours. C1QA, C1QC, C3, C4A, C4B were not available in this dataset.

Supplementary Figure 11. Effect of different types of interferon on complement gene expression in fibroblasts (GSE67737). C1R and C1S had the highest expression among complement genes. There was a discreet overexpression of C1R and C1S after treatment with IFNg.

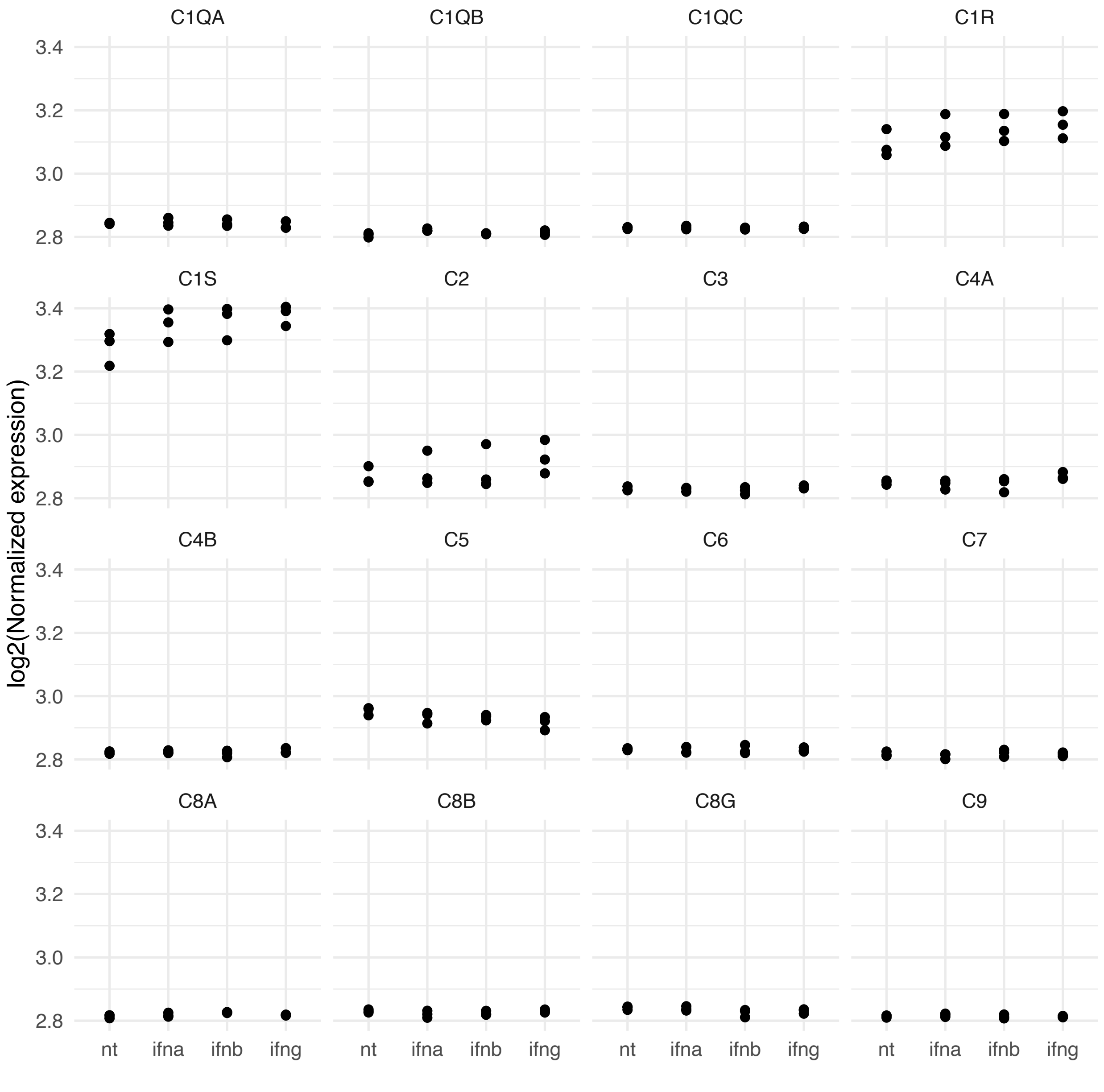

Scaled to the maximum value of all genes. nt: untreated; ifna: treated with 1000IU/mL of IFNA for 10h; ifnb_100u: treated with 1000IU/mL of IFNB for 10h; ifng_100u: treated with 1000IU/mL of IFNG for 10h.

Supplementary Figure 12. Effect of different types of interferon on complement gene expression in fibroblasts (GSE50954). C1R and C1S had the highest expression among complement genes. There was a discreet overexpression of C1R and C1S after treatment with IFNg.

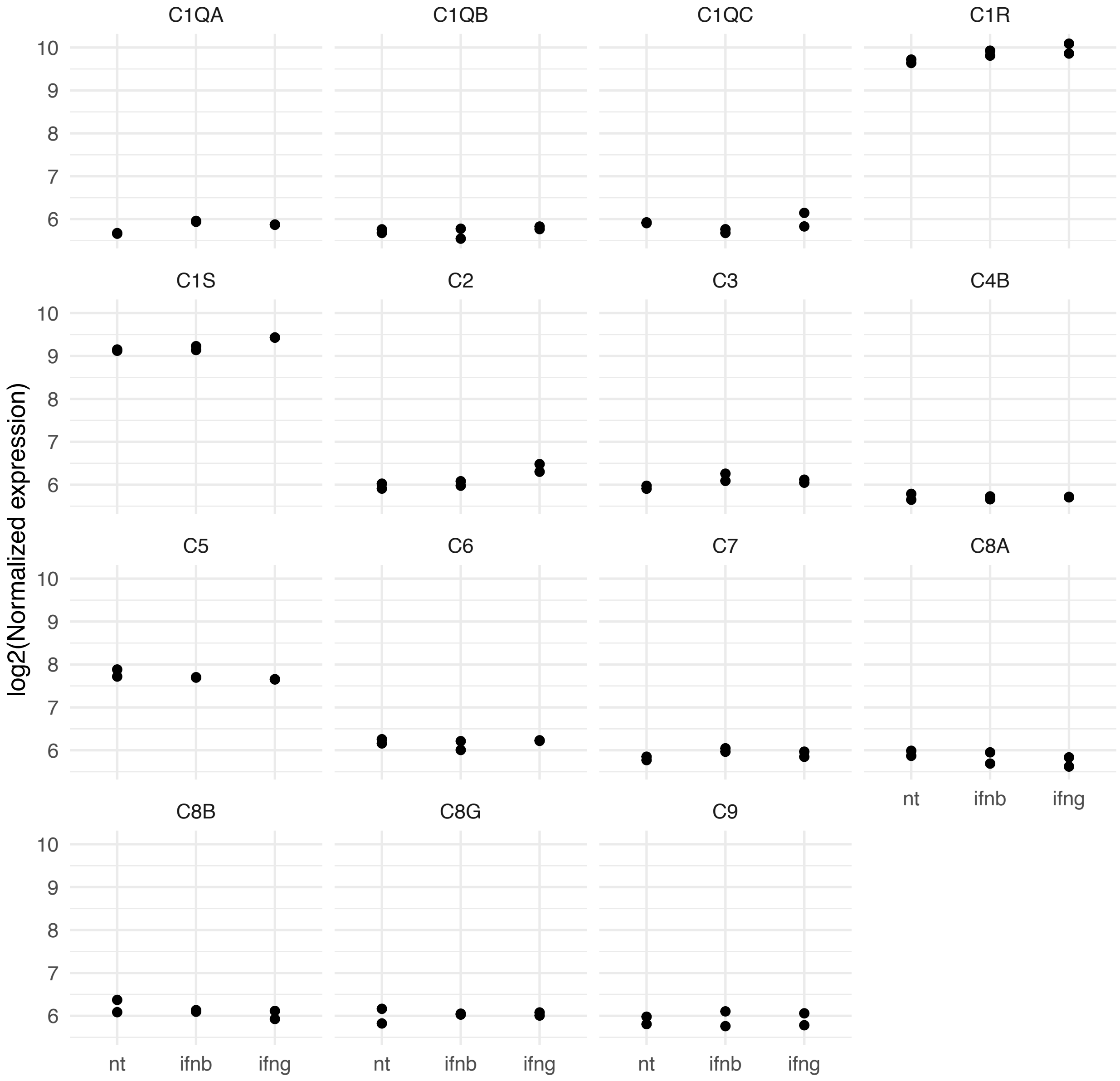

Scaled to the maximum value of all genes. nt: untreated; ifnb: treated with IFNB for 6h; ifng: treated with IFNG for 6h.
